# Positive transcription elongation factor b (P-TEFb) is a therapeutic target in human multiple myeloma

**DOI:** 10.18632/oncotarget.19761

**Published:** 2017-08-01

**Authors:** Yu Zhang, Liang Zhou, Yun Leng, Yun Dai, Robert Z. Orlowski, Steven Grant

**Affiliations:** ^1^ Division of Hematology/Oncology, Department of Medicine, Virginia Commonwealth University and The Massey Cancer Center, Richmond, VA, USA; ^2^ Department of Hematology, Beijing Chaoyang Hospital of Capital Medical University, Beijing, China; ^3^ Cancer Center, The First Hospital of Jilin University, Changchun, China; ^4^ Department of Myeloma and Lymphoma, MD Anderson Cancer Center, Houston, TX, USA; ^5^ Virginia Institute of Molecular Medicine, Virginia Commonwealth University, Richmond, VA, USA; ^6^ Department of Biochemistry, Virginia Commonwealth University, Richmond, VA, USA; ^7^ Department of Pharmacology Virginia Commonwealth University, Richmond, VA, USA

**Keywords:** P-TEFb, bortezomib resistance, myeloma, MCL-1, CDK inhibitors

## Abstract

The role of the positive RNA Pol II regulator, P-TEFb (positive transcription elongation factor b), in maintenance of the anti-apoptotic protein Mcl-1 and bortezomib (btz) resistance was investigated in human multiple myeloma (MM) cells. Mcl-1 was up-regulated in all MM lines tested, including bortezomib-resistant lines, human MM xenograft mouse models, and primary CD138^+^ MM cells. Mcl-1 over-expression significantly reduced bortezomib lethality, indicating a functional role for Mcl-1 in bortezomib resistance. MM cell lines, primary MM specimens, and murine xenografts exhibited constitutive P-TEFb activation, manifested by high CTD (carboxy-terminal domain) S2 phosphorylation, associated with a) P-TEFb subunit up-regulation i.e., CDK9 (42 and 55 kDa isoforms) and cyclin T1; and b) marked CDK9 (42 kDa) T186 phosphorylation. In marked contrast, normal hematopoietic cells failed to exhibit up-regulation of p-CTD, CDK9, cyclin T1, or Mcl-1. CDK9 or cyclin T1 shRNA knock-down dramatically inhibited CTD S2 phosphorylation and down-regulated Mcl-1. Moreover, CRISPR-Cas CDK9 knock-out triggered apoptosis in MM cells and dramatically diminished cell growth. Pan-CDK e.g., dinaciclib or alvocidib and selective CDK9 inhibitors (CDK9i) recapitulated the effects of genetic P-TEFb disruption. CDK9 shRNA or CDK9 inhibitors significantly potentiated the susceptibility of MM cells, including bortezomib-resistant cells, to proteasome inhibitors. Analogously, CDK9 or cyclin T1 knock-down or CDK9 inhibitors markedly increased BH3-mimetic lethality in bortezomib-resistant cells. Finally, pan-CDK inhibition reduced human drug-naïve or bortezomib-resistant CD138^+^ cells and restored bone marrow architecture *in vivo*. Collectively, these findings implicate constitutive P-TEFb activation in high Mcl-1 maintenance in MM, and validate targeting the P-TEFb complex to circumvent bortezomib-resistance.

## INTRODUCTION

Multiple myeloma (MM) is an accumulative disease of mature plasma cells, and despite recent advances in treatment, including the introduction of effective new drugs such as proteasome inhibitors and immunomodulatory agents, it remains largely incurable. Like numerous neoplastic disorders, MM is characterized by dysregulation of members of the Bcl-2 family of pro- and anti-apoptotic proteins. In particular, the multi-domain anti-apoptotic protein Mcl-1 (myeloid cell leukemia-1) is frequently over-expressed in MM [1]. Mcl-1 is a short-lived protein (T1/2 ~ 2-3 hr) [2] which cooperates with other anti-apoptotic proteins e.g., Bcl-xL to bind and inactivate pro-apoptotic effectors such as Bax thereby preventing apoptosis [3]. The short half-life of Mcl-1 raises the possibility that efficient transcriptional machinery may be required for its constitutive *de novo* expression in MM. Indeed, studies employing antisense or knock-down strategies have shown that Mcl-1 plays a critical functional role in MM cell survival [4, 5]. In addition, proteasome inhibitors such as bortezomib, by blocking Mcl-1 degradation, induce Mcl-1 accumulation, which may contribute to resistance to such agents [6, 7]. Collectively, these considerations provide a strong rationale for targeting Mcl-1 in MM, particularly in the setting of proteasome inhibitor resistance.

Eukaryotic protein-coding gene transcription is regulated at multiple levels, including by the activity of the p-TEFb (positive transcription elongation factor b) CDK9/cyclinT complex, which phosphorylates the carboxy-terminal domain (CTD) of RNA Polymerase II (RNAPII) on serine residues 2 and 5 of RNA Pol II. The latter permits productive elongation and co-transcriptional modifications of transcripts necessary for effective transcription [8]. P-TEFb is a holoenzyme CDK9/cyclin T complex which is reciprocally regulated by negative (N-TEF) and positive elongation factors (P-TEF) [8]. Cyclin-dependent kinase inhibitors represent a class of agents that disrupt the function of cyclin-dependent kinases (CDKs), proteins which act in conjunction with cyclins to allow progression of cells through the cell cycle [9]. Although it was initially assumed that the antitumor effects of these agents stemmed from blocking cell cycle progression, it has subsequently been shown that a sub-set of CDK inhibitors (e.g., those that inhibit CDK9) can also act through a transcriptional mechanism by down-regulating the expression of various short-lived proteins such as Mcl-1 and p21^CIP1^ [10, 11]. Flavopiridol (alvocidib), a pan-CDK inhibitor and potent inhibitor of p-TEFb [9], was the first CDK inhibitor to enter the clinical arena. In preclinical studies, alvocidib demonstrated marked activity against MM cells, in part related to its ability to down-regulate Mcl-1 [9]. In clinical trials, single-agent alvocidib activity in MM has been limited, although activity when combined with other agents (e.g., bortezomib) has been reported [12]. Such considerations have led to the developments of second-generation CDK inhibitors such as dinaciclib (SCH727965), a highly potent inhibitor of CDKs 1,2, 5, and 9 which has shown significant activity in pre-clinical studies against several tumor types [13–16], and more recently activity in MM [17, 18].

Currently, the role of CDK9 as a therapeutic target in MM has not been definitively validated, nor has the relationship between perturbations in the CDK9/cyclin T axis and increased Mcl-1 expression been systematically examined, particularly in the context of bortezomib resistance. Here we report that in MM cells, increased expression as well as activation of cyclin T and CDK9 play critical functional roles in Mcl-1 maintenance, including in the setting of bortezomib resistance, and that targeting components of the P-TEFb pathway pharmacologically or genetically potently down-regulate Mcl-1 expression and promote cell death, particularly in the presence of proteasome inhibitors or BH3-mimetics. The present results also argue that MM cells, in contrast to their normal counterparts, are specifically addicted to an activated P-TEFb complex for survival, providing a basis for therapeutic selectivity. Collectively, these findings provide a theoretical foundation for targeting the P-TEFb complex in proteasome inhibitor-resistant MM.

## RESULTS

### Mcl-1 is constitutively expressed in MM *in vitro* and *in vivo* and confers bortezomib resistance

**Figure 1:**
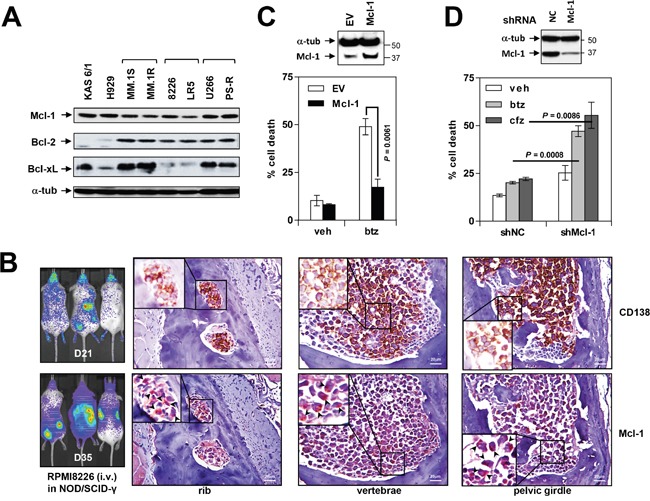
Mcl-1 is highly expressed in MM cell lines *in vitro* and *in vivo* and is associated with bortezomib resistance **(A)** Immunoblotting analysis was performed as described in Methods to profile basal expression levels of Mcl-1, Bcl-2 and Bcl-xL in untreated human MM cell lines. Lanes were loaded with 30 μg of protein; α-tubulin controls were assayed to ensure equivalent loading and transfer. **(B)** NOD/SCID-γ mice were injected intravenously with 5×10^6^ luciferase-labeled RPMI8226 cells and images captured at day 21 and 35 with the Xenogen IVIS 200 imaging system. In addition, rib, vertebrae and pelvic girdle sections were stained with anti-CD138 and Mcl-1 antibodies, after which images were obtained with an IX71-Olympus inverted system microscope. Scale bar = 20 μm. **(C)** U266 cells were stably transfected with an Mcl-1 construct. Cells were then exposed to 5 nM bortezomib (btz) for 24 hr, followed by flow cytometry to monitor the percentage of apoptotic (Annexin V^+^) cells. Values represent the means ± S.D. for three experiments performed in triplicate. **(D)** PS-R cells were stably transfected with constructs encoding shRNA targeting Mcl-1 (shMcl-1) or scrambled sequence as a negative control (shNC). Cells were then exposed to 15 nM btz or 25 nM carfilzomib for 24 hr, followed by flow cytometry to determine the percentage of apoptotic (annexin V^+^) cells. Values represent the means ± S.D. for three experiments performed in triplicate.

Bcl-2 family profiling of eight MM cell lines revealed robust and relatively uniform Mcl-1 expression in all lines (Figure 1A), including PS-R (bortezomib-resistant U266) cells previously shown to exhibit modest increases in Mcl-1 but marked reductions in Bim expression [19]. Bcl-2 expression was also observed in all but two of the lines, whereas Bcl-xL expression was considerably more variable. Injection of NOD/SCID-γ mice with luciferase-labeled RPMI8226 cells demonstrated extensive dissemination of MM by day 21 and 35 (Figure 1B, left panel). Staining of bone sections with labeled anti-CD138 and Mcl-1 antibodies revealed extensive co-localization in the marrow (Figure 1B, right panels), demonstrating that MM cells are characterized by pronounced Mcl-1 expression both *in vitro* and *in vivo*.

To assess the role of Mcl-1 over-expression in bortezomib resistance, U266 cells were stably transfected with a Mcl-1 construct. U266/Mcl-1 cells expressed a marked increase in Mcl-1 protein compared to empty-vector controls (Figure 1C, upper panel). Notably, U266/Mcl-1 cells were significantly more resistant to bortezomib (5 nM) than their empty-vector counterparts (P < 0.01; Figure 1C, lower panel), indicating that Mcl-1 down-regulation may enhance bortezomib sensitivity in MM. Conversely, Mcl-1 knock-down in PS-R cells significantly sensitized MM cells to the proteasome inhibitors bortezomib and carfilzomib (cfz; P < 0.01 in each case; Figure 1D).

### MM cell lines exhibit high levels and activation of the P-TEFb apparatus

**Figure 2:**
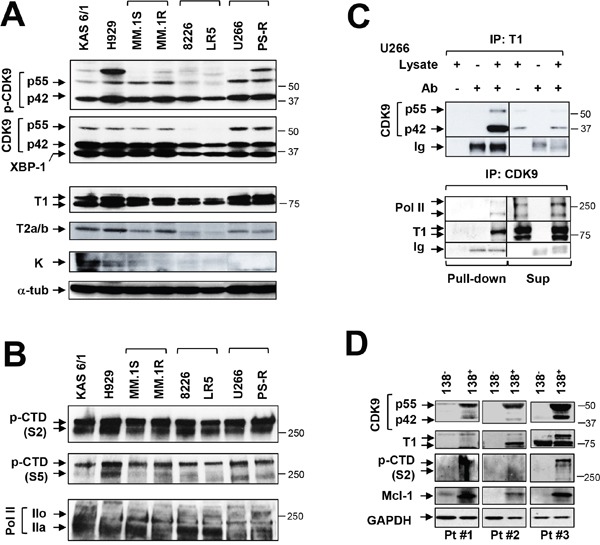
MM cell lines and primary MM but not normal cells display P-TEFb activation and high Mcl-1 expression **(A)** Immunoblotting analysis was performed to profile basal levels of p-CDK9, CDK9, XBP-1, cyclin T1, cyclin T2a/b and cyclin K in the indicated untreated human MM cell lines. Lanes were loaded with 30 μg of protein; α-tubulin controls were assayed to ensure equivalent loading and transfer. Duplicate experiments yielded equivalent results. **(B)** Phosphorylated (serine-2 and 5, CTD) forms of RNA pol II and pol II were monitored by immunoblotting analysis in human MM cell lines as described in A. **(C)** U266 cells were lysed in 1% CHAPs buffer and subjected to immunoprecipitation. IP was carried out in pull-down and supernatant sections individually with cyclin T1 antibody, and immunoblotted for CDK9; reverse IP was performed with CDK9 antibody, and immunoblotted for cyclin T1, Pol II. Replicate experiments yielded equivalent results. **(D)** Primary CD138^+^ cells were isolated from primary bone marrow samples obtained from three MM patients (Pt #1-3). The CD138^+^ cells and their CD138^-^ counterparts were subjected to immunoblot analysis for basal levels of CDK9, cyclin T1, phosphorylated (serine-2, CTD) RNA pol II and Mcl-1 as described above. Blots were probed for GAPDH expression to confirm equivalent loading and transfer.

Multiple components of the P-TEFb transcriptional regulatory apparatus were then examined in MM cell lines and primary cells. Constitutive phosphorylation of CDK9 (42 kDa) was detected in all cell lines, whereas p-CDK9 (55 kDa) expression was more variable (Figure 2A). In accord with these findings, total CDK9 (42 kDa) was abundantly expressed in all lines, whereas total CDK9 (55 kDa) exhibited greater variability. Consistent with evidence of its role in myelomagenesis [20], XBP-1 was also robustly expressed in all cell lines.

Expression of cyclin T1 was very pronounced in all eight cell lines, whereas expression of cyclin T2a/b was variable and cyclin K minimal (Figure 2A). These phenomena were associated with prominent and uniform S2 phosphorylation of CTD accompanied by more variable S5 phosphorylation (Figure 2B). Finally, both total RNAP II and the phosphorylated forms, were clearly expressed across cell lines (Figure 2B). Concordant results were obtained in tumor tissues extracted from mice inoculated with U266 cells (data not shown).

In addition, immunoprecipitation studies in U266 cells revealed a marked association between p55 and p42 CDK9 and cyclin T1 (Figure 2C upper panel). Reverse IP studies (CDK9 IP; cyclin T1 WB) confirmed these findings and demonstrated association of CDK9 with RNAPII and cyclin T1 (Figure 2C, lower panel). Virtually identical findings were obtained in bortezomib-resistant PS-R cells (Supplementary Figure 1) and in U266 cells extracted from flank tumors in NSG mice (data not shown). Collectively, these and the preceding findings indicate that MM cells are characterized by pronounced constitutive expression and activation of the transcriptional regulatory apparatus e.g., expression of cyclin T1, expression and phosphorylation of CDK9, association of CDK9/cyclin T1/RNAPII, and S2 phosphorylation/activation of the carboxy-terminal domain of RNAPII.

### Primary MM but not normal cells display P-TEFb activation and high Mcl-1 expression

The preceding studies were then extended to include three primary CD138^+^ MM specimens and their CD138^-^ normal counterparts. Notably, all three CD138^+^ samples exhibited robust expression of p42 and p55 CDK9, whereas no or minimal expression was detected in normal CD138^-^ cells (Figure 2D). Similarly, cyclin T1 was clearly expressed in CD138^+^ but not CD138^-^ cells. In addition, S2 p-CTD in CD138^+^ cells was clearly observed in two specimens, faintly discerned in the second, but was absent in CD138^-^ cells. Significantly, expression of Mcl-1 was very pronounced in CD138^+^ MM cells, but essentially absent in their normal counterparts (Figure 2D). These findings support the notion that MM cells, but not their normal counterparts, are dependent upon an active transcriptional apparatus to maintain high Mcl-1 levels required for survival, providing a theoretical basis for targeting P-TEFb in the selective killing of MM cells.

### Genetic or pharmacologic disruption of the transcriptional regulatory apparatus down-regulates Mcl-1 in bortezomib-sensitive or -resistant MM cells

**Figure 3:**
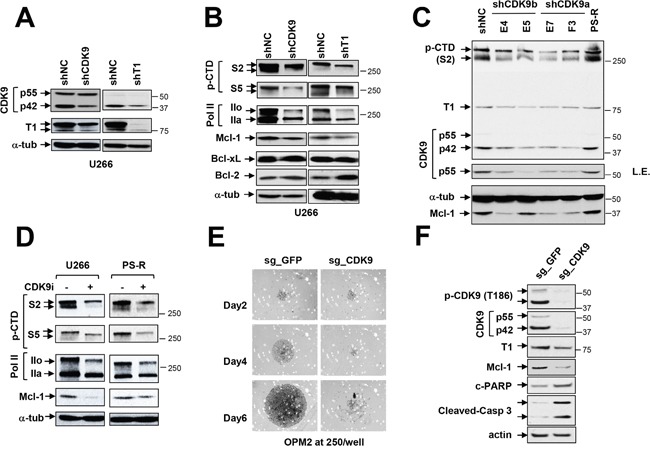
Genetic or pharmacologic disruption of the transcriptional regulatory apparatus down-regulates Mcl-1 in bortezomib-sensitive or -resistant MM cells **(A-B)** U266 cells were stably transfected with constructs encoding shRNA targeting CDK9 (shCDK9) or cyclin T1 (shT1) or scrambled sequence as a negative control (shNC). Immunoblotting analysis was performed to profile basal levels of CDK9, cyclin T1, phosphorylated forms (serine-2 and 5, CTD) of RNA pol II, Mcl-1, Bcl-2, and Bcl-xL in shCDK9 and shT1 cells as described previously. α-tubulin controls were assayed to ensure equivalent loading and transfer. Replicate experiments yielded equivalent results. **(C)** PS-R (bortezomib-resistant U266) cells were stably transfected with constructs encoding shRNA targeting CDK9 (shCDK9a or shCDK9b), and subjected to immunoblotting analysis to monitor basal levels of CDK9, cyclin T1, phosphorylated (serine-2, CTD) RNA pol II and Mcl-1. Lanes were loaded with 30 μg of protein; α-tubulin controls were assayed to ensure equivalent loading and transfer. Duplicate experiments yielded equivalent results. L.E. indicates long exposure. **(D)** U266 and PS-R cells were treated with CDK9i 15 μM for 24 hr, after which expression of phosphorylated (serine-2 and 5, CTD) RNA pol II, pol II, and Mcl-1 was monitored by immunoblotting analysis as in C. α-tubulin controls were assayed to ensure equivalent loading and transfer. Duplicate experiments yielded equivalent results. **(E)** OPM2 MM cells were infected with lentivirus encoding Cas9 and sgRNA targeting GFP or CDK9. After infection and selection with puromycin (1.5 mg/ml, 48 hr), cells were seeded in a 48-well round-bottom plate (250 cells per well), and images were obtained on day 2, 4 and 6. Images were obtained with an IX71-Olympus research inverted system microscope at 40× magnification. **(F)** Protein extracts were obtained from non-targeting and sgCDK9 cells, and immunoblotting analysis performed to monitor expression of CDK9 (55 and 42 kDa), cyclin T1, Mcl-1, and cleaved PARP and caspase 3. Lanes were loaded with 30 μg of protein; β-actin controls were assayed to ensure equivalent loading and transfer. Duplicate experiments yielded equivalent results.

To assess the functional significance of individual components of the P-TEFb complex in Mcl-1 maintenance, an shRNA strategy was employed. To this end, U266 cells were genetically engineered to express CDK9 or cyclin T1 shRNA (Figure 3A). In these cells, CDK9 or cyclin T knock-down modestly but discernibly down-regulated S2 CTD and RNA Pol IIo (the phosphorylated form) (Figure 3B). These changes were accompanied by Mcl-1 down-regulation but no significant changes in expression of Bcl-2 or Bcl-xL were observed.

Attempts were then made to extend these findings to bortezomib-resistant PS-R cells using multiple CDK9a or b shRNA clones (E4, E5, E7, F3; Figure 3C). All CDK9 shRNA clones displayed diminished S2 CTD phosphorylation compared to empty-vector or untransfected PS-R cells. As observed with U266 cells, CDK9 knock-down was associated with down-regulation of S2 CTD phosphorylation, and clear dephosphorylation of p42 and p55 CDK9, the latter more apparent with long exposure intervals (L.E.; Figure 3C). As also observed in U266 cells, CDK9 knock-down resulted in marked reductions in Mcl-1 expression.

The effects of a specific CDK9 inhibitor (CDK9i) [21] on these events were then evaluated. Exposure of U266 or bortezomib-resistant PS-R cells to CDK9i resulted in sharp reductions in S2 CTD and S5 CTD phosphorylation (Figure 3D). This was accompanied by down-regulation of phosphorylated RNA Pol IIo and diminished expression of Mcl-1. Together, these findings argue that both cyclin T1 and CDK9 play significant functional roles in maintaining Mcl-1 expression in bortezomib-resistant or -sensitive cells, and that the consequences of genetic disruption of the P-TEFb apparatus can be recapitulated by pharmacological CDK9 inhibitors, including in bortezomib-resistant MM cells.

### CRISPR-Cas CDK9 knock-out dramatically diminishes MM cell proliferation and survival

To confirm the effects of CDK9 disruption more definitively, OPM2 MM expressing CDK9 CRISPR-Cas knock-out cells were generated. Bright-field microscopic images of wells inoculated with CDK9 knock-out cells revealed a dramatic reduction in cell growth compared to non-targeting controls (Figure 3E). Consistent with these findings, CDK9 knock-out cells displayed a marked reduction in levels of pCDK9 and p42 or p55 compared to non-targeting controls, accompanied by diminished expression of Mcl-1and sharp increases in cleaved PARP and caspase-3 (Figure 3F). These findings provide further evidence of a critical role for CDK9 in MM cell survival and growth.

### CDK inhibitors block CTD phosphorylation, down-regulate Mcl-1, and induce apoptosis in bortezomib-sensitive or -resistant cells

**Figure 4:**
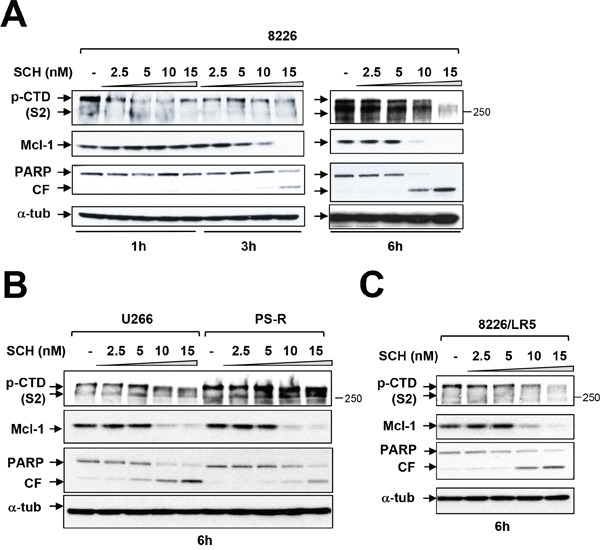
Dinaciclib (SCH) induces apoptosis in various MM cells in association with Pol II inhibition and Mcl-1 downregulation **(A)** Logarithmically growing RPMI8226 cells were exposed to 2.5 nM to 15 nM dinaciclib (SCH) for 1, 3, and 6 hr, after which protein extracts were obtained and subjected to immunoblot analysis to monitor the expression of phosphorylated forms (serine-2 and 5, CTD) of RNA pol II, Mcl-1, and PARP as described previously. Lanes were loaded with 30 μg of protein; α-tubulin controls were assayed to ensure equivalent loading and transfer. Duplicate experiments yielded equivalent results. CF = cleavage fragment. **(B)** U266 and PS-R (bortezomib-resistant U266) cells were exposed to 2.5 nM to 15 nM SCH for 6 hr. After treatments, immunoblotting analysis was carried out to monitor phosphorylated form (serine-2, CTD) of RNA pol II, Mcl-1, and PARP cleavage fragment (CF). **(C)** RPMI8226/LR5 (melphalan-resistant) cells were treated the same as B. for 6 hr. After treatments, immunoblotting analysis was carried out to monitor the phosphorylated (serine-2, CTD) RNA pol II, Mcl-1, and PARP cleavage (CF). Each lane was loaded with 30 μg of protein; α-tubulin controls were assayed to ensure equivalent loading and transfer.

The effects of more clinically relevant and more broadly acting CDK inhibitors on these events was then examined. Exposure of RPMI8226 cells to very low concentrations (e.g., 10-15 nM) of the CDK1,2,5, and 9 inhibitor dinaciclib diminished S2 CTD phosphorylation as early as 3 hr of exposure, accompanied by Mcl-1 down-regulation, which was essentially complete by 6 hr, as well as marked PARP cleavage (Figure 4A). Of note, the early (e.g., 6 hr) dephosphorylation of CTD and Mcl-1 down-regulation by dinaciclib was unaccompanied by changes in cell cycle distribution (Supplementary Figure 2). Parallel results were observed in bortezomib-sensitive U266 or -resistant (PS-R) cells (Figure 4B) as well as RPMI8226/LR5 (melphalan-resistant) cells (Figure 4C) or bortezomib-resistant RPMI8226/VR cells [22] (data not shown). Together, these findings indicate that clinically relevant CDK9 inhibitors block RNA Pol II CTD phosphorylation in MM cells at early intervals, resulting in Mcl-1 down-regulation and cell death, and that analogous events occur in bortezomib-resistant cells.

### Genetic or pharmacologic CDK9 inhibition promotes proteasome inhibitor lethality in bortezomib-resistant MM cells

**Figure 5:**
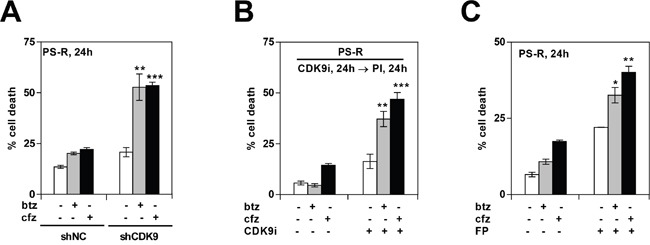
Genetic or pharmacologic CDK9 inhibition promotes proteasome inhibitor (PI) lethality in bortezomib-resistant MM cells **(A)** PS-R (bortezomib-resistant U266) cells were stably transfected with constructs encoding shRNA targeting CDK9 (shCDK9) or scrambled sequence (shNC). Cells were then treated with 15 nM bortezomib (btz) or 25 nM carfilzomib (cfz) for 24 hr, after which cell death was analyzed by flow cytometry after staining with 7-AAD. Significantly greater than values for control cells: ** = P < 0.01; *** = P < 0.001. **(B)** PS-R cells were treated with CDK9i (15 μM) for 24 hr, followed by exposure to 15 nM btz or 25 nM cfz for an additional 24 hr. Cell death (7-AAD) was analyzed by flow cytometry. ** = P < 0.01; *** = P < 0.001. **(C)** PS-R cells were treated with btz (15 nM) or cfz (25 nM) with or without alvocidib (FP; 150 nM) for 24 hr, and then analyzed by flow cytometry to determine the percentage of apoptotic cells. Significantly greater than control; * = P < 0.05; ** = P < 0.01.

Previous studies have shown that CDK inhibitors can potentiate the activity of proteasome inhibitors in various malignant hematopoietic cells [23]. The ability of pharmacologic or genetic disruption of CDK9 to enhance the activity of such agents in bortezomib-resistant MM cells was then examined. As shown in Figure 5A, bortezomib-resistant PS-R cells stably expressing CDK9 shRNA were significantly more sensitive to the lethal effects of bortezomib or carfilzomib (cfz) compared to their control counterparts (P < 0.01 or 0.001). Analogously, exposure of bortezomib-resistant PS-R cells to the CDK9-selective inhibitor CDK9i significantly increased bortezomib or carfilzomib lethality (P < 0.01 or 0.001; Figure 5B). Comparable results were obtained in bortezomib-resistant PS-R cells with the clinically relevant pan-CDK inhibitor alvocidib (Figure 5C). Analogously, similar results were obtained in parental U266 cells when CDK inhibitors was combined with low concentrations of proteasome inhibitors (Supplementary Figure 3). Together, these findings indicate that genetic or pharmacologic interruption of CDK9 enhances proteasome inhibitor lethality in MM cells, including in cells exhibiting bortezomib resistance.

### Pharmacologic CDK9 inhibition promotes BH3-mimetic inhibitor lethality in bortezomib-resistant MM cells

**Figure 6:**
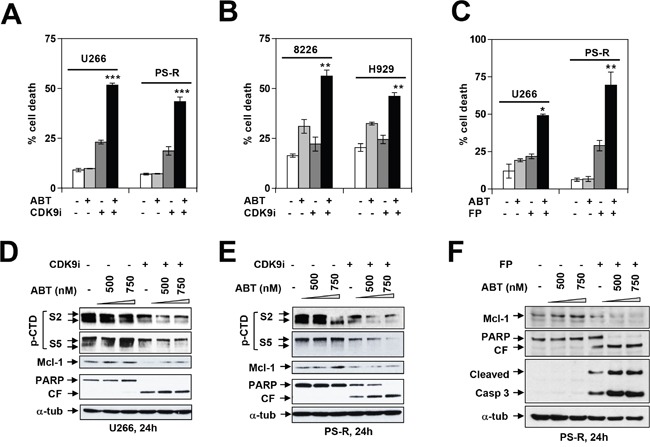
Dinaciclib or a CDK9-specific inhibitor potentiate BH3 mimetics lethality in MM cells in association with Pol II inhibition and Mcl-1 downregulation U266 and PS-R (bortezomib-resistant U266) cells **(A)** or RPMI8226 and H929 cells **(B)** were exposed (24 hr) to 500 nM ABT-737 with or without 20 μM CDK9i, followed by flow cytometry to determine the percentage of apoptotic cells *** = P < 0.001, significantly greater than values for ABT alone; ** = P < 0.01. **(C)** Parallel studies were performed with 100 nM alvocidib (FP) and 750 nM ABT-737. * = P < 0.05; ** = P < 0.01. Immunoblotting analysis was carried out to monitor expression of phosphorylated (serine-2 and 5, CTD) RNA pol II, Mcl-1, and PARP cleavage in U266 cells **(D)** and PS-R cells **(E)** exposed (24 hr) to the indicated concentrations of ABT-737 and CDK9i (20 μM). **(F)** PS-R cells were exposed to ABT ± FP for 24 hr as described in (C), after which immunoblotting analysis was carried out to monitor expression of Mcl-1 and cleavage of PARP and caspase 3 in PS-R cells.

Previous studies have shown that CDK inhibitors potentiate the lethal effects of BH3-mimetics in various malignant hematopoietic cells e.g., AML or lymphoma [24–26]. Consequently, parallel studies were performed with BH3-mimetics in MM cells, including those resistant to bortezomib. As shown in Figure 6A, co-administration of the CDK9i significantly increased the lethal effects of the BH3-mimetic ABT-737 in both parental (U266) as well as bortezomib-resistant (PS-R) MM cells. Comparable results were observed in RPMI8226 and H929 cells (Figure 6B), and in bortezomib-sensitive or -resistant (PS-R) U266 cells exposed to ABT-737 in conjunction with alvocidib (Figure 6C). Immunoblot analysis revealed that combined ABT-737/CDK inhibitor exposure in U266 or bortezomib-resistant cells resulted in diminished CTD phosphorylation, Mcl-1 down-regulation, and increased PARP cleavage (Figure 6D-6F). Finally, concordant results were obtained in bortezomib-sensitive or -resistant MM cells exposed to dinaciclib in combination with the BH3-mimetic HA-14 [27] (Supplementary Figure 4A-4C). Similar results were obtained in U266 cells or when alvocidib was used in conjunction with ABT-737 (data not shown). Collectively, these findings indicate that pharmacologic CDK9 interruption significantly lowers the threshold for BH3-mimetic lethality in both bortezomib-sensitive or -resistant MM cells.

### Genetic interruption of the P-TEFb complex potentiates BH3-mimetic lethality in MM cells

**Figure 7:**
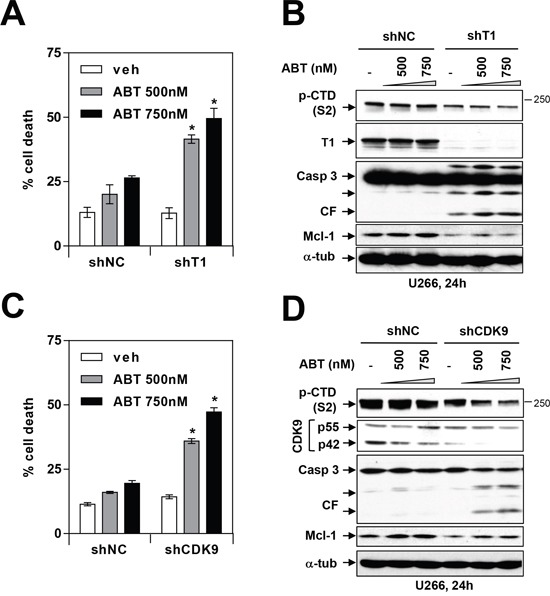
Genetic inhibition of CDK9 or cyclin T1 potentiates BH3-mimetic lethality in MM cells **(A** and **C)** U266 cells were stably transfected with constructs encoding shRNA targeting cyclin T1 (A) or shRNA targeting CDK9 (C) or scrambled sequence (shNC). Cells were then treated with 500-750 nM ABT-737 for 24 hr, and cell death was analyzed by flow cytometry after staining with 7-AAD. * = P < 0.05, significantly greater than control. **(B** and **D)** Following treatment as described above, immunoblotting analysis was carried out to monitor serine-2 phosphorylation of the CTD of RNA pol II, cyclin T1, CDK9, cleaved caspase 3, and Mcl-1. Each lane was loaded with 30 μg of protein; α-tubulin controls were assayed to ensure equivalent loading and transfer.

To confirm the effects of these pharmacologic CDK9 inhibitors, parallel studies were performed in U266 cells expressing Cyclin T1 or CDK9 shRNA. As shown in Figure 7A and 7C, Cyclin T1 shRNA or CDK9 shRNA cells were significantly more sensitive to ABT-737 than their control counterparts. This effect was accompanied by diminished CTD phosphorylation (S2), marked Mcl-1 down-regulation, and pronounced caspase 3 cleavage (Figure 7B and 7D). These findings indicate that genetic disruption of the P-TEFb complex recapitulate the actions of CDK9 inhibitors and in so doing, lowers the apoptotic threshold of MM cells exposed to BH3-mimetics.

### CDK inhibitors suppress growth and bone marrow infiltration of bortezomib-resistant MM cells *in vivo*

**Figure 8:**
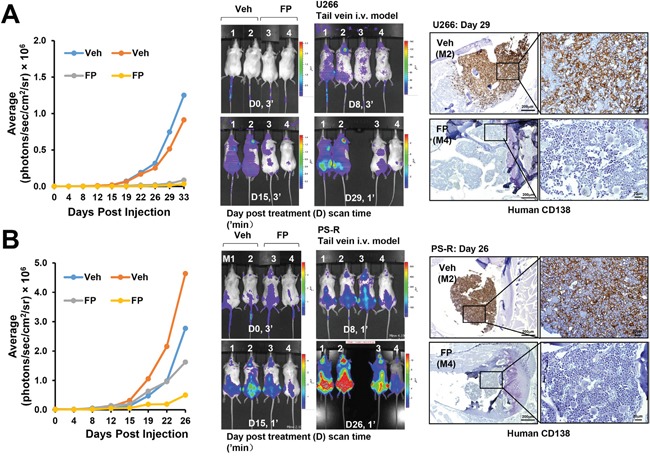
A CDK9 inhibitor (alvocidib) suppresses the growth of both drug-naïve and bortezomib-resistant cells in a tail-vein i. v. systemic murine model and dramatically diminishes human CD138+ MM cells in the BM **(A** and **B)** NOD/SCID-γ mice were injected intravenously via tail vein with 5×10^6^ U266 cells (A) or PS-R (B) stably expressing luciferase. After luciferase signals were visible (e.g., 14 days after injection of tumor cells), alvocidib (FP; 5 mg/kg) was administered via intraperitoneal (i.p.) injection daily 5 days a week, 4 weeks; n = 2 per group. Tumor growth was quantified by average luciferase activity (photons/sec/cm^2^/sr × 10^6^, left panels), and represented (middle panels). Long bone tissue sections (right panels) from vehicle, and FP-treated animals were stained by immunohistochemistry with human CD138 antibody. Images were obtained with an IX71-Olympus research inverted system microscope. Scale bar = 200 or 20 μm.

The impact of CDK inhibition against bortezomib-sensitive or resistant cells was then assessed *in vivo*. NOD/SCD-γ mice were injected via tail vein with luciferase-labeled U266 cells and treated with alvocidib (FP; 5 mg/kg) or vehicle control for about one month. As shown in Figure 8A, left and middle panels, animals treated with alvocidib displayed a clear reduction in tumor signal compared to those treated with vehicle controls. In addition, bone marrow sections stained with labeled antibodies to human CD138 (brown stain) revealed virtual elimination of MM cells in the marrows (Figure 8A, right panel). Notably, very similar results were obtained in bortezomib-resistant PS-R cell-bearing mice e.g., reduction by alvocidib of tumor signal (Figure 8B, left and middle panels), and diminution of CD138^+^ cells in the marrow (Figure 8B, right panel). A dual-flank model was then employed in which animals were inoculated with U266 and PS-R cells in the right and left flanks respectively. A reduction in signal over a 22-day period compared to untreated controls was observed in alvocidib-treated mice for both U266 and PS-R cells (Supplementary Figure 5A). In addition, immunoblotting analysis of tumor tissue obtained from mice inoculated with U266 cells revealed a marked reduction in p-CTD (S2) and a modest but discernible reduction in Mcl-1 expression in alvocidib-treated mice (Supplementary Figure 5B). Finally, H&E-stained marrow sections revealed loss of marrow architecture by MM cell infiltration in vehicle-treated animals, but substantial restoration of normal architecture in the marrows with alvocidib treatment in both U266- and PS-R-bearing mice (Supplementary Figure 6A and 6B). Together, these findings argue that disruption of the transcriptional regulatory apparatus e.g., by CDK inhibitors can antagonize MM cell survival *in vivo* as well as *in vitro*, and that such activity can occur in bortezomib-resistant as well as -sensitive cells.

## DISCUSSION

Although CDKIs were initially developed to exploit deregulated cell cycle proliferation characteristic of transformed cells [28, 29], other CDKI mechanisms of action of have been described, including disruption of DNA repair [30]. In addition, a subset of CDKIs e.g., those inhibiting CDK7 or CDK9, interfere with the P-TEFb machinery necessary for active transcription of diverse proteins [11, 31]. The present studies demonstrate that the P-TEFb transcriptional regulatory apparatus, and particularly CDK9, provide an important survival mechanism for MM cells, including those resistant to proteasome inhibitors such as bortezomib, at least in part through maintenance of Mcl-1 expression. While Mcl-1 has been recognized as a critical survival factor in MM [1, 5, 7], the mechanism(s) by which this protein is maintained has not been systematically examined in these cells. It is noteworthy that high Mcl-1 expression in both cell lines, including those sensitive or resistant to bortezomib, as well as primary MM cells was associated with constitutive activation of RNA Pol II, accompanied by high basal levels of both CDK9 and cyclin T1. Because the Mcl-1 protein has a short half-life (e.g., 2-3 hr) [2], active transcription is required to replenish protein depleted by proteasomal degradation [32]. It is therefore tempting to speculate that in MM cells, the high levels of expression of components of the P-TEFb complex, as well as their constitutive activation, are required for Mcl-1 maintenance and cell survival. In our previous study, we demonstrated that inhibiting CDK9/pTEFb can diminish MCL-1 transcription [23]. In support of this notion, a specific CDK9 inhibitor, as well as more broadly acting CDK inhibitors that also target CDK9 e.g., dinaciclib and alvocidib [12] blocked CTD phosphorylation, down-regulated Mcl-1 expression, and triggered cell death. Significantly, knock-down of either CDK9 or cyclin T1, essential for RNA Pol II activation [33], essentially recapitulated the actions of specific or more broadly acting CDK inhibitors, arguing that CDK9 disruption represents a critical functional target in this setting. Although it has been reported that Bcl-2, a protein with a relatively long half-life [34] may also represent an important survival factor for MM cells [35], pharmacologic or genetic disruption of the P-TEFb apparatus killed MM cells without down-regulating this protein, arguing that reductions in Mcl-1 played a primary role in this setting. Moreover, as observed in the case of dinaciclib, down-regulation of Mcl-1 and cell death induction substantially preceded (e.g., within 6 hr) cell cycle arrest, supporting the notion that the cell death and cell cycle inhibitory effects of this agent are dissociable.

Disruption of proteasome function can lead to Mcl-1 accumulation [32], a phenomenon that has been associated with proteasome inhibitor resistance [7]. Consistent with these findings, ectopic expression of Mcl-1 in MM cells significantly reduced bortezomib sensitivity. It is important to note that highly bortezomib-resistant MM cells (PS-R) exposed to CDK inhibitors (e.g., dinaciclib or alvocidib) exhibited marked disruption of P-TEFb activation, reductions in Mcl-1 expression comparable to those of bortezomib-sensitive cells, and equivalent degrees of cell death. Moreover, as in the case of parental cells, CDK9 knock-down effectively reduced RNA Pol II activation, reflected by S2 and S5 CTD dephosphorylation, accompanied by apoptosis. Together, these findings suggest that bortezomib-resistant MM cells, like their sensitive counterparts, may retain their reliance on Mcl-1 for survival, and that targeting P-TEFb represents a potentially effective strategy in this setting.

Mcl-1 is highly expressed in MM cells, presumably through activation of an IL-6-dependent signaling axis [36]. Among cell cycle regulatory proteins, Cyclin D1 is also frequently overexpressed in this disorder [37]. We also observed high Mcl-1 expression in all MM cell lines, as well as in primary CD138^+^ specimens. However, to the best of our knowledge, this is the first report demonstrating high expression of cyclin T1 and CDK9 in MM, including in primary MM cells, and constitutive activation of RNA Pol II. Such findings suggest that MM cells exhibit priming of the transcriptional apparatus in order to maintain sufficiently high Mcl-1 levels required for survival. Interestingly, normal CD138^-^ bone marrow cells expressed low levels of cyclin T1, phospho-CTD, and Mcl-1. This raises the possibility that normal cells, in contrast to their MM counterparts, are not dependent upon high Mcl-1 expression for survival, and as a consequence, do not require an active transcriptional apparatus. It also raises the possibility that targeting CDK9 or other components of the transcriptional machinery involving P-TEFb, may selectively target MM cells while sparing normal hematopoietic cells.

Previous studies have shown that CDKIs (e.g., roscovitine) increase the lethality of the BH3-mimetic ABT-737, which inhibits Bcl-2/xL but not Mcl-1, in human leukemia cells [38]. Similarly, CDKIs also increase proteasome inhibitor activity in AML cells [39], while a CDK4/6 inhibitor, which does not inhibit transcription, potentiated bortezomib lethality in MM cells [40]. Here, we observed that addition to inducing cell death, CDK9 inhibitors promoted the anti-myeloma activity of both proteasome inhibitors as well as BH3-mimetics, and that these effects were observed in both bortezomib-sensitive or -resistant cells. Furthermore, in both cell types, knock-down of cyclin T1 or CDK9 recapitulated the effects of CDK inhibition in promoting P-TEFb inactivation, Mcl-1 down-regulation, and cell death. Such findings, along with the observation that Mcl-1 knock-down analogously increased proteasome inhibitor lethality, argue that these actions reflect disabling of Mcl-1 cytoprotective activities. However, there are alternative mechanisms by which CDK inhibition may promote cell death in multiple myeloma and other cell types including a) enhanced DNA damage [41]; b) disruption of cytoprotective autophagy [42]; and c) disabling of cytoprotective ER stress responses [43], both of which can be triggered by BH3-mimetics and proteasome inhibitors [23, 44]. It is therefore possible that these actions may contribute to the activity of these combinations. Given the limited activity of single-agent CDKIs to date in MM [17], it is possible that the primary role of these agents will ultimately lie in rational combination therapy, particularly in the setting of proteasome inhibitor-resistant disease.

Results from *in vivo* models demonstrated widespread dissemination of MM cells, accompanied by marked CD138^+^ and Mcl-1 co-localization in the marrows of inoculated mice, consistent with a postulated Mcl-1 requirement for the *in vivo* survival of MM cells. Notably, CDK9 inhibitor treatment of mice inoculated with bortezomib-sensitive or -resistant MM cells yielded approximately equivalent reductions in tumor burden, clearing of marrow, and restoration of marrow architecture. These actions were accompanied by dephosphorylation of S2 CTD and Mcl-1 down-regulation, as observed *in vitro*. Together, these observations argue that disruption of P-TEFb complex may represent a viable therapeutic strategy in MM both *in vivo* as well as *in vitro*, and that it may be effective against at least some forms of proteasome inhibitor resistance.

In summary, the present findings provide a theoretical basis for targeting the P-TEFb apparatus in MM, and suggest that the increased expression and constitutive activation of components of this complex necessary for Mcl-1 maintenance may offer a window for therapeutic selectivity. They also raise the possibility that MM cells resistant to proteasome inhibitors may retain their dependence upon Mcl-1 for survival, and thus be vulnerable to strategies targeting the transcriptional regulatory machinery. Although limited trials of CDK9 inhibitors have not yet established clear single-agent activity in MM [17, 45], it is possible that sub-optimal scheduling or pharmacokinetics may have limited the efficacy of such agents to date [46]. However, it is likely that the ultimate value of such agents may lie in rational combinations e.g., with BH3-mimetics or proteasome inhibitors, particularly in the setting of resistant disease [23, 44] or disease characterized by pronounced P-TEFb activation. In this regard, clinical studies suggest that dinaciclib may have some, albeit limited single-agent activity in MM [17], and initial trials combining dinaciclib with bortezomib show some promise [30, 45]. Finally, the present findings support the evaluation in bortezomib-resistant MM of alternative P-TEFb antagonists, including more specific CDK9 inhibitors [47, 48], or CDK7 inhibitors, recently shown to be active in pre-clinical AML models [31]. Moreover, recent studies suggest that bromodomain antagonists interfere with P-TEFb [49], and therefore might similarly be effective in the setting of bortezomib resistance. Accordingly, studies designed to test these hypotheses are currently underway.

## MATERIALS AND METHODS

### Human multiple myeloma (MM) cell lines

Human MM U266, H929, RPMI8226, and IL-6-dependent KAS 6/1 cell lines were obtained from ATCC and maintained as before [42]. Dexamethasone-sensitive (MM.1S) and -resistant (MM.1R) cell lines were provided by Dr. Steven T. Rosen (Northwestern University, Chicago, IL, USA). Melphalan-resistant (LR5) RPMI8226 sublines were maintained as before [50]. To establish human MM cells adaptive resistance to bortezomib, U266 cells were continuously cultured in gradually increasing concentrations of bortezomib (beginning at 0.5 nM and increasing in stepwise increments of 0.2 nM) to 20 nM. Another bortezomib-resistant RPMI8226 (8226/V10R) subline was similarly established and maintained in 10 nM bortezomib. All experiments utilized logarithmically growing cells (3-5×10^5^ cells/ml).

### Isolation of primary MM cells

Fresh bone marrow (BM) samples were obtained with informed consent according to the Declaration of Helsinki and VCU IRB approval from patients with MM undergoing routine diagnostic aspirations. CD138^+^ and CD138^-^ cells were isolated from bone marrow (BM) samples using a MACS magnetic separation technique (Miltenyi Biotech, Auburn, CA, USA) as per the manufacturer's instructions. The purity (> 90%) and viability (> 95%) of CD138^+^ fractions was determined by flow cytometry and trypan blue exclusion, respectively. Isolated cells were maintained in RPMI1640 medium containing 10% FBS.

### Reagents

The Bcl-2/Bcl-xL/Bcl-w antagonist ABT-737 were purchased from Chemie-Tek, Indianapolis, IN. The pan-CDK inhibitors SCH727965 (Dinaciclib), and flavopiridol (Alvocidib) were purchased from Selleck (Houston, TX, USA). The selective CDK9 inhibitor II (4-(3,5-Diamino-1H-pyrazol-4-ylazo)-phenol) and the Bcl-2 antagonist HA14-1 were purchased from Calbiochem/EMD Chemicals/Millipore and BioMol/Alexis/Enzo respectively. Bortezomib and carfilzomib were purchased from Chemie-Tek.

Drugs were dissolved in sterile DMSO, aliquoted and stored at -80°C. In all experiments, final DMSO concentrations did not exceed 0.1%.

### RNA interference

SureSilencing shRNA plasmids targeting human CDK9 (shCDK9) (GenBank accession number NM_001261; GGTCAAGTTCACGCTGTCTGA), cyclin T1 (shcyclin T1) (accession number NM_001240: TCGTGTCCCTCATTCGAAACT or scrambled sequence as negative control (shNC; GGAATCTCATTCGATGCATAC) were purchased from SABioscience (Frederick, MD, USA). The CDK9 shRNA is specific for the isoform of p42 CDK9 based on the target sequence, Cells were stably transfected with these constructs using the Amaxa Nucleofector device with Cell Line Specific Nucleofector Kit C (Amaxa GmbH, Cologne, Germany) as per the manufacturer's instructions. Clones with down-regulated CDK9 or cyclin T1 were selected with 400 μg/ml G418.

### CRISPR/cas9 plasmids

Construction of lenti-CRISPR/CAS9 vectors targeting transcriptional CDK was performed following the protocol associated with the backbone vector (49535, Addgene) [51]. The following sequences were chosen from the published literature [52].

GFP (fwd: CACCGGGGCGAGGAGCTGTTC ACCG;

rv: AAACCGGTGAACAGCTCCTCGCCCC),

CDK9 (fwd: CACCGGCACCGCAAGACCGGC CAGA

rv: AAACTCTGGCCGGTCTTGCGGTGCC).

### Virus infection

Lentiviruses were generated in Phenix cells by transfecting cells with packaging DNA plus tet-on-pLKO or lenti-CRISPR vectors. Typically 2 μg vector DNA, 1.5 μg psPAX2, and 1 μg pMD2-VSVG, 10 μl FuGENE® 6 Transfection Reagent (Roche, IN) were used. FuGENE® 6 Transfection Reagent was first added to serum-free medium (Opti-MEM® I Reduced-serum medium). The solution was mixed and incubated for 5 minutes, after which DNA was added to the FuGENE® 6 Transfection Reagent/medium, which was then mixed and incubated for an additional 15 minutes. Mixtures were added to Phenix cells e.g., 5×10^6^ cells seeded in one 10 cm dish one day earlier. Viral supernatant was collected two and three days after transfection, filtered through 0.45 μm membranes, and added to target cells in the presence of polybrene (8 μg/ml, Millipore). Puromycin (1.5 μg/ml) was used to treat cells for two days for selection, which eliminated all cells in the uninfected control group.

### Cell cycle analysis

Cell cycle analysis by propidium iodide (PI) staining in the presence of RNase A was performed by flow cytometry (FCM) using the Modfit LT2.0 software (Verity Software House, Topsham, ME, USA) as described previously [53].

### Analysis of cell death

Apoptosis was evaluated by flow cytometry utilizing Annexin V-FITC/PI staining as before [53]. Cell death were assessed by 7-AAD as before [53].

### Immunoblotting analysis

Samples from whole-cell lysates were prepared, and 30 μg of protein per condition were subjected to immunoblotting analysis as previously described [53]. Where indicated, the blots were re-probed with antibodies against β-actin, α-tubulin or GAPDH (EMD/Millipore/Sigma, Billerica, MA, USA) to ensure equal loading and transfer of proteins. Primary antibodies included: Caspase 3, Mcl-1 (BD-Pharmingen, San Diego, CA, USA); caspase 9, cleaved caspase 3 (Asp175), cleaved caspase 9 (Asp315), Bcl-xL, cleaved PARP (Asp214) and p-CDK9 (T186) (Cell Signaling, Beverly, MA, USA); RNA Polymerase II (EMD/Millipore/Sigma, Billerica, MA, USA), RNA Polymerase II (H5) (the phosphoserine 5 form of pol II), and RNA Polymerase II (H14) (the phosphoserine 2 form of pol II) (BioLegend, Dedham, MA, USA); human Bcl-2 oncoprotein (DAKO, Carpinteria, CA, USA); PARP (Enzo, Plymouth Meeting, PA, USA); CDK9, cyclin T1, cyclinT2a/b, cyclin K, and XBP-1 (Santa Cruz Biotech, Santa Cruz, CA, USA).

### Immunoprecipitation

Co- immunoprecipitation analysis was performed to evaluate interactions between CDK9 and cyclin T1. Briefly, cells were lysed in CHAPS buffer (150 mM NaCl, 10 mM HEPES pH7.4, protease inhibitors, and 1% CHAPS) and 200 μg of protein per condition was incubated with 1 μg anti-cyclin T1 or anti-CDK9 overnight at 4°C. 20 μl/condition of Dynabeads (Dynal, Oslo, Norway) were then added and incubated for an additional 4 h. Samples were centrifuged at 500×g for 5 min to remove the supernatants. Bead-bound protein was eluted from washed pellets by vortexing and boiling in 20 μl sample buffer. The samples were then separated by SDS-PAGE and subjected to immunoblotting analysis as described above.

### Animal studies

All animal studies were IACUC approved and performed in accordance with AAALAC, USDA, and PHS guidelines. For the dual-side flank murine model, NOD/SCID-γ mice (Jackson Laboratories) were inoculated s.c. in opposite flanks with 5×10^6^ bortezomib-resistant PS-R and U266 cells. For the orthotopic murine model, NOD/SCID-γ mice were injected i.v. with either 5×10^6^ U266 or PS-R cells stably transfected with constructs encoding luciferase. After tumors were visible, FP (5 mg/kg) was administrated (i.p.) 5 days a week. Control animals received equal volumes of vehicle.

### Histology

Bones were excised for histological examination and fixed in neutral buffered formaldehyde (10%) overnight at 4°C. Samples were washed with water and decalcified in 10% EDTA (pH7.4) for 14 days, until they lost normal structural rigidity. The bones were then embedded in paraffin bocks and 5 μm sections were cut. Sections were subsequently stained with hematoxylin and eosin (H&E), and immunohistochemically processed using anti-CD138 or Mcl-1 antibodies, and evaluated by histopathology. Sections were visualized and images captured using an Olympus IX71 Inverted System Microscope with a DP73 Digital Camera.

### Statistical analysis

Values represent the means ± SD for at least three independent experiments performed in triplicate. The significance of differences between experimental variables was determined using the One-way ANOVA with Tukey–Kramer Multiple Comparisons Test and Student's t test. *P* < 0.05 was considered significant.

## SUPPLEMENTARY FIGURES



## Abbreviations

Flavopiridol, FP: Alvocidib
SCH727965, SCH: Dinaciclib
btz: Bortezomib
cfz: Carfilzomib
H&E: Hematoxylin and eosin
