# Supplementary material for: Positive transcription elongation factor b (P-TEFb) is a therapeutic target in human multiple myeloma
Source: Oncotarget. 2017 Aug 1;8(35):59476–91. doi: 10.18632/oncotarget.19761 (PMC5601747; doi:10.18632/oncotarget.19761)
Supplement: Supplementary file 1 [file oncotarget-08-59476-s001.pdf]

Positive transcription elongation factor b (P-TEFb) is a therapeutic target in human multiple myeloma

SUPPLEMENTARY MATERIALS

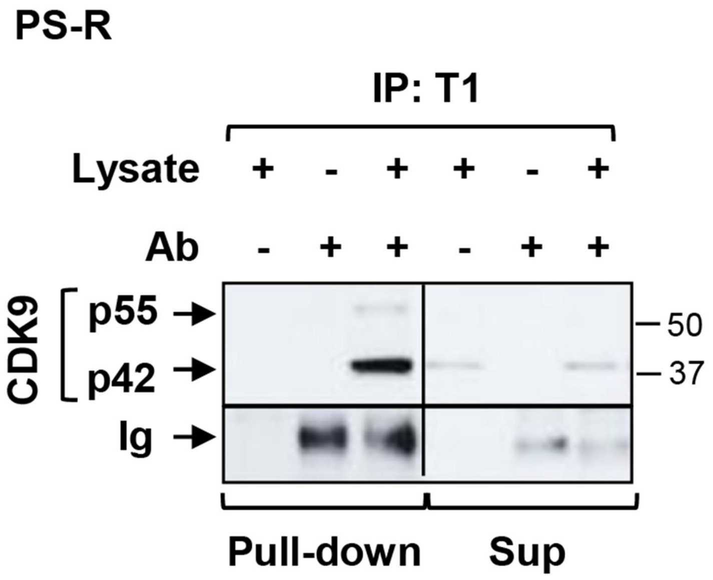

Supplementary Figure 1: Cyclin T1 co-immunoprecipitates with CDK9 in bortezomib-resistant MM cells. PS-R (bortezomib-resistant U266) cells were lysed in 1% CHAPs buffer and subjected to immunoprecipitation. IP was carried out in pull-down and supernatant sections individually with cyclin T1 antibody, and immunoblotted for CDK9.

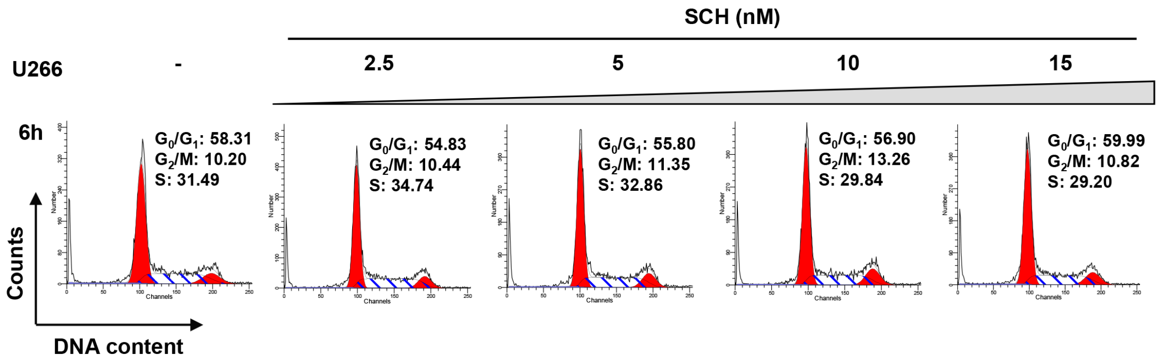

Supplementary Figure 2: Dinaciclib (SCH) kills MM cells prior to disruption of the cell cycle. U266 cells were exposed to SCH (2.5 nM to 15 nM) for 6 hr, after which cell cycle analysis was performed by PI staining and flow cytometry. Values indicate the percentage of cells in G0/G1, S, or G2/M. Duplicate experiments yielded equivalent results.

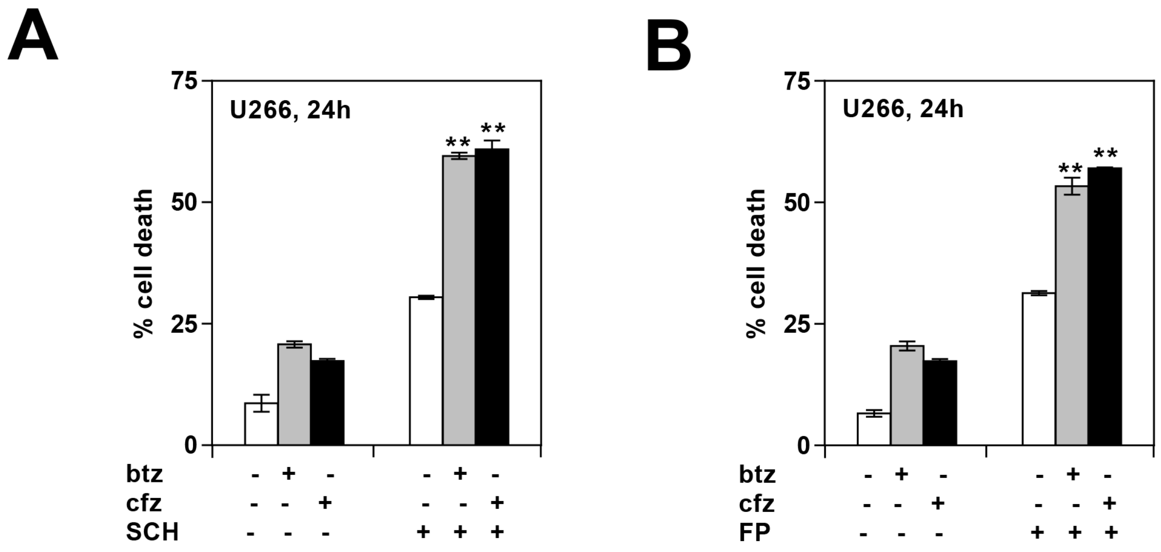

Supplementary Figure 3: Genetic or pharmacologic CDK9 inhibition promotes proteasome inhibitor (PI) lethality in bortezomib-sensitive MM cells. **(A)** U266 cells were treated with btz (2 nM) or cfz (5 nM) with or without dinaciclib (SCH; 7 nM) for 24 hr, and then analyzed by flow cytometry to determine the percentage of apoptotic cells. Cell death (7-AAD) was analyzed by flow cytometry. Significantly greater than control; ** = $P < 0.01$. **(B)** U266 cells were treated with btz (2 nM) or cfz (5 nM) with or without alvocidib (FP; 150 nM) for 24 hr, and then analyzed by flow cytometry to determine the percentage of apoptotic cells. Significantly greater than control; ** = $P < 0.01$.

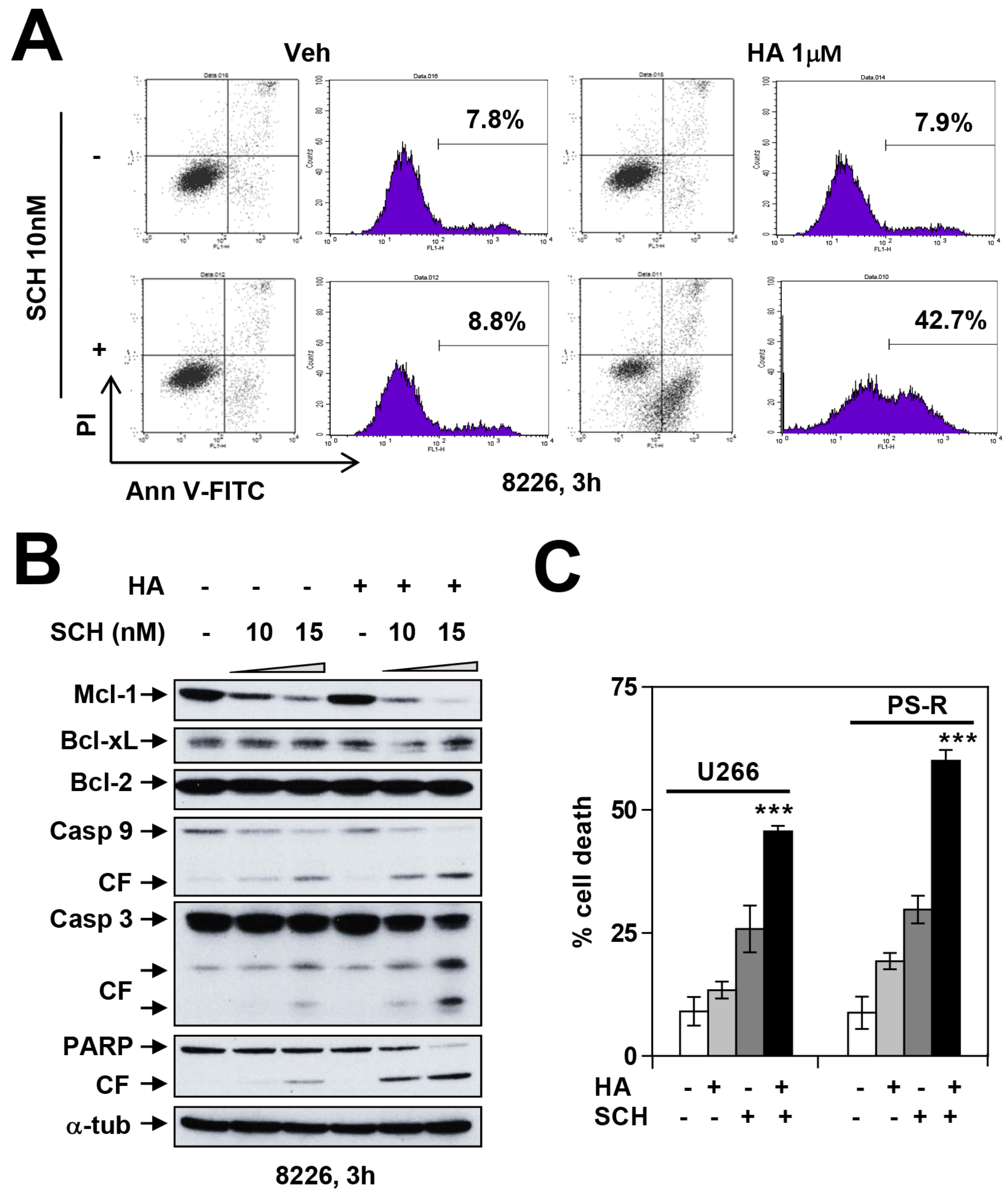

Supplementary Figure 4: Dinaciclib markedly increases HA-14-induced cell death in both sensitive and bortezomib-resistant MM cells. (A) RPMI8226 cells were exposed to dinaciclib (SCH; 10 nM) with or without HA-14 (1μM) for 3 hr, followed by flow cytometry to monitor the percentage of apoptotic (Annexin V+) cells. **(B)** RPMI8226 cells were exposed to SCH 10 nM or 15 nM with or without HA-14 1μM for 3 hr, after which cell extracts were immunoblotted to monitor expression of Mcl-1, Bcl-xL, Bcl-2, and cleaved caspase 9, caspase 3, and PARP. α-tubulin controls were assayed to ensure equivalent loading and transfer. **(C)** U266 and PS-R cells were exposed to SCH 10 nM with or without HA-14 (1μM) for 24 hr, after which cell death was analyzed by flow cytometry after staining with 7-AAD. Values represent means ± S.D. for three replicate determinations. *** = $P < 0.001$, significantly greater than values for individual agents.

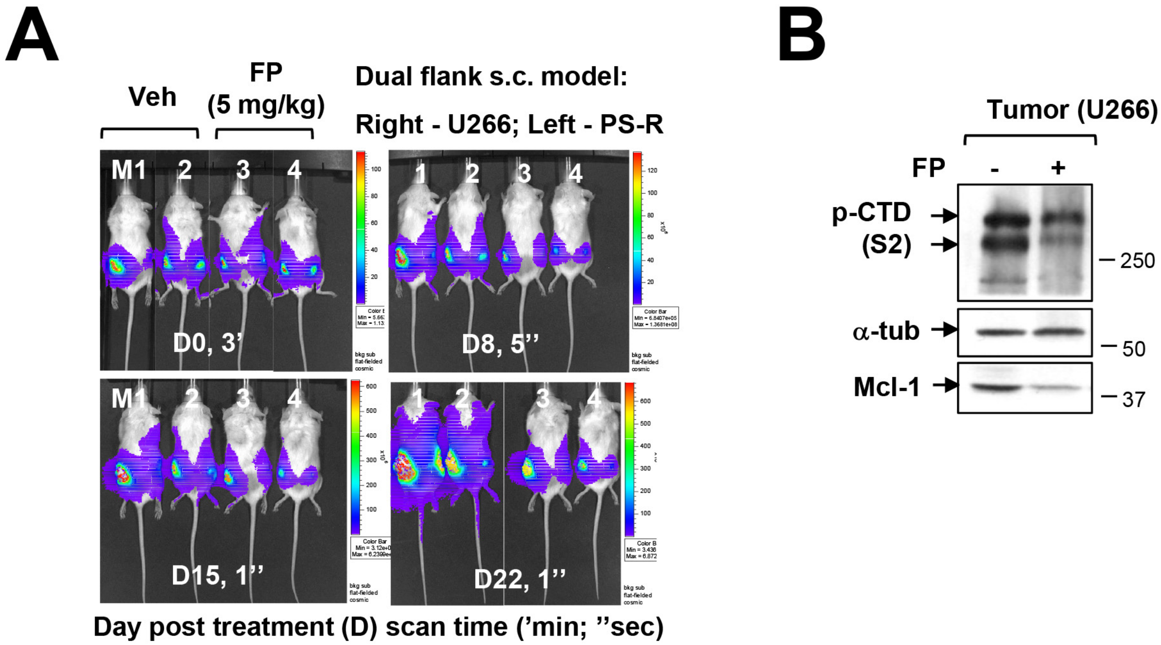

Supplementary Figure 5: Alvocidib (FP) suppresses tumor growth of either drug-naïve or bortezomib-resistant cells in a dual flank murine model in association with Pol II inhibition and Mcl-1 downregulation. **(A** and **B)** NOD/SCID-γ mice were subcutaneously inoculated in the right rear flank with 5×10^6 U266 cells stably expressing luciferase, and the left rear flank with 5×10^6 PS-R (bortezomib-resistant U266) cells stably expressing luciferase. Treatment was initiated after luciferase activity was detected (7 days after injection of tumor cells). Mice were treated with FP (5 mg/kg) via intraperitoneal (i.p.) injection daily 5 days a week for 4 weeks. Control animals were administered equal volumes of vehicle. Tumor growth was monitored every other day after i.p. injection with 150 mg/kg luciferin using the Xenogen IVIS 200 imaging system. When tumor size reached 2,000 mm^3, all mice were euthanized, and tumors removed and subjected to immunoblot analysis for phosphorylated CTD (serine-2) and Mcl-1 levels. Each lane was loaded with 30 μg of protein, and α-tubulin controls were assayed to ensure equivalent loading and transfer.

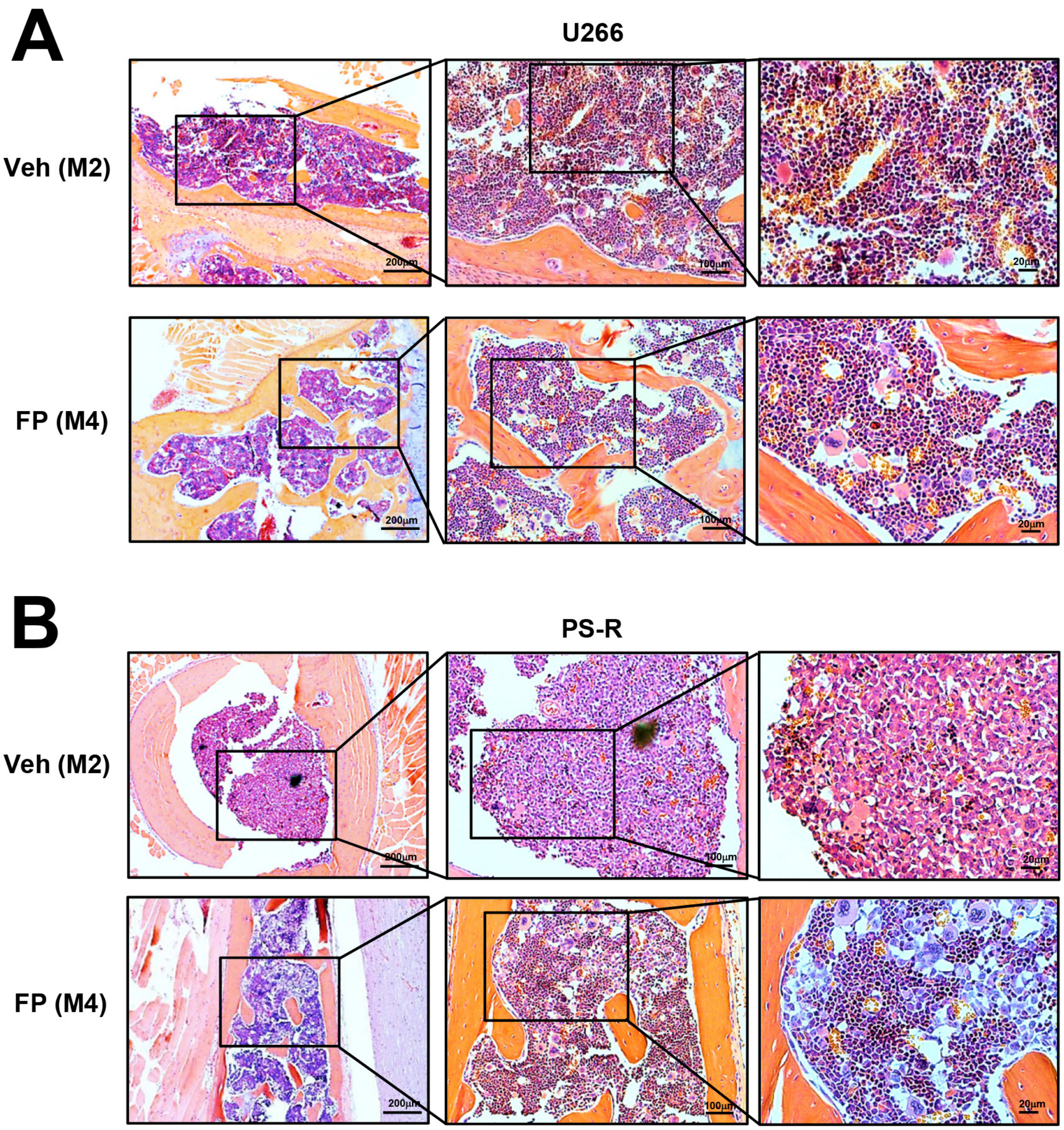

Supplementary Figure 6: Alvocidib (FP) restores the BM structure. Long bone tissue sections from vehicle, and FP-treated animals (Figure 8A, systemic models) were stained by H&E, and images obtained with an IX71-Olympus inverted system microscope. Scale bar = 200 μm; 100 μm; 20 μm.
